# YAP1 Upregulates Cytoskeleton Regulator ARHGEF1 and Tissue Regeneration Factor NEDD9 in a Multiplex Proteomic Study

**DOI:** 10.3390/neurolint18050096

**Published:** 2026-05-21

**Authors:** Dinesh Devadoss, Juliet Akkaoui, Arti Vashist, Adriana Yndart Arias, Adel Nefzi, Madepalli K. Lakshmana

**Affiliations:** 1 Department of Cellular and Molecular Medicine, Herbert Wertheim College of Medicine, Florida International University, 11200, SW 8th Street, University Park, Miami, FL 33199, USA; ddevados@fiu.edu (D.D.); jakka001@fiu.edu (J.A.); avashist@fiu.edu (A.V.); ayndarta@fiu.edu (A.Y.A.); 2 FIU Center for Translational Science, 11350 SW Village Parkway, Port Saint Lucie, FL 34987, USA; anefzi@fiu.edu

**Keywords:** YAP1 Alzheimer’s disease, multiplex array, autophagy, proteomics, cytoskeleton, NEDD9, ARHGEF1, NENF, senescence

## Abstract

**Background/Objectives**: Yes-associated protein 1 (YAP1) is a transcriptional cofactor that coordinates the complex interplay between cell proliferation, survival, differentiation, metabolism, biomechanics, and tissue regeneration. Previous studies have shown that YAP1 activity is reduced during aging, and replacing YAP1 function has been shown to rejuvenate old cells by mitigating senescence and its associated inflammation. **Methods**: As YAP1 is now confirmed to exert a profound regenerative influence on multiple organs, we wanted to gain more insight into the molecular signature of YAP1 expression relevant to brain cells. Since proteomics is a very powerful tool for discoveries, we generated SH-SY5Y cells stably expressing GFP-YAP1 and screened 8000 human proteins using multiplex arrays that utilize biotin-label-based antibody arrays. **Results**: We found YAP1 expression in astrocytes, microglia, neuronal and neuroblastoma cell lines, as well as human neurons. Importantly, YAP1 protein levels were significantly reduced selectively in the nuclear fractions of the brains of patients with Alzheimer’s disease (AD) relative to normal control (NC) subjects. The screen resulted in the identification of 283 differentially expressed proteins. In line with YAP1’s known role in the regulation of actin and cytoskeleton, we found a 2.53-fold upregulated level of Rho guanine nucleotide exchange factor 1 (ARHGEF1), a guanine nucleotide exchange factor (GEF) for the RhoA GTPase, which is crucial for dendritic spine regulation. A 6.19-fold upregulated level of NECAP endocytosis-associated 2 (NECAP2), the highest known increase for any protein in this screen, plays an essential role in clathrin-mediated endocytosis. Most importantly, another upregulated protein was Neudesin Neurotrophic Factor (NENF) (3.07-fold increase), also known as Neudesin, which primarily acts as a neurotrophic factor, and it promotes neuronal survival, enhances cell proliferation, and neurogenesis in neural progenitor cells. Neural Precursor Cell Expressed, Developmentally Down-Regulated 9(NEDD9) levels were also upregulated by 2.46-fold, and it affects neuronal cell number and synaptic connections through its role in neurite formation. However, it should be noted that these proteomic results are preliminary in nature as they are derived from single-sample data. The upregulated levels of ARHGEF1 and NEDD9 were confirmed by immunoblots. We also found a drastic reduction in the levels of p16INK4a, a marker of senescence. **Conclusions**: Thus, the anti-senescence effect of YAP1 may be mediated through p16INK4a, which in turn may be crucial for YAP1’s regenerative functions through NENF and NEDD9.

## 1. Introduction

Yes-associated protein 1 (YAP1) acts as both a coactivator and a corepressor in the downstream Hippo signaling pathway critical for organ size control and tumor suppression through restricting proliferation and promoting apoptosis depending on the cell type and physiological context [1,2,3,4]. In the Hippo signaling pathway, phosphorylation of YAP1 by large tumor suppressor kinase 1 and 2 (LATS1/2) prevents its nuclear translocation, thereby regulating the expression of its target genes. This transcriptional regulation of gene expression also requires TEA domain family member (TEAD) transcription factors. YAP1 can regulate organ size because YAP1 can sense various types of mechanical cues, including membrane tension, cell stretching, and cell stiffness via actin stress fibers, which initiates YAP1 nuclear import to activate the downstream transcriptional program [5,6,7]. However, although YAP1 is now a well-established transcriptional cofactor, it does not itself have any DNA-binding activity [8,9,10].

Importantly, genome-wide analysis of YAP/TAZ-binding targets has identified promoters that drive the expression of critical genes, including those that encode Yamanaka pluripotency factors crucial for stem cell potency, such as SRY (sex-determining region Y)-box 2, also known as SOX2, homeobox protein NANOG, Octamer-binding transcription factor 4 (OCT4), and myelocytoma (MYC) [11]. This enables YAP1 to play important roles in cell proliferation, differentiation, and tissue regeneration [12,13,14]. Because experimental activation of YAP/TAZ in the mouse can promote regeneration of vital organs such as heart, liver, and intestine in old or diseased mice, artificial YAP/TAZ activation is considered an excellent strategy in stimulating organ repair and regeneration as demonstrated in mice [15,16,17]. Recent evidence also indicates that YAP1 signaling plays an important role in aging and senescence [18,19,20]. Thus, YAP1 has been shown to inhibit senescence of multiple cell types, including mesenchymal stem cells [18], fibroblasts [19], hepatic stem cells [21], glioma cells [20], as well as hippocampal astrocytes of the aging mice and mouse models of Alzheimer’s disease(AD) [22]. Induction of premature astrocyte senescence following selective knockout of YAP1 further established the crucial role of YAP1 in astrocyte senescence, most likely through Cyclin-dependent kinase 6 (CDK6) signaling [22]. Further, suppression of the YAP pathway in senescent cells has been shown during DNA damage-induced senescence [23] and oncogene-induced senescence [24]. Interestingly, a recent study further demonstrated that stromal YAP activity decreases during aging, and sustaining YAP function rejuvenates old cells by reducing senescence and its associated inflammation [25]. Taken together, these data imply that YAP1 gain of function inhibits senescence and the senescence-associated secretory phenotype (SASP), but on the contrary, its loss of function exaggerates senescence, suggesting that YAP1 is a bona fide therapeutic target to reduce senescence.

The crucial role of YAP1 in the brain relevant to neurodegenerative diseases has also been demonstrated in multiple models. YAP1/TAZ was shown to be required to promote myelination during the differentiation and maturation of Schwann cells [26,27], YAP1 maintains the dynamics of transactive response DNA binding protein 43 (TDP-43) condensates and antagonizes TDP-43 pathological aggregates [28], age-dependent YAP1 reduction upregulates the nuclear receptor 4A1 (Nr4a1)–Ak strain-transforming (AKT)–glycogen synthase kinase-3 beta (GSK-3β) axis and contributes to AD pathology [29], YAP1 alleviates sepsis-associated encephalopathy by inhibiting hippocampus ferroptosis via maintaining homeostasis in mitochondrial dynamics [30], and lack of YAP1 sensitizes brain during Hypoxic–Ischemic Injury [31]. Furthermore, the Hippo/YAP signaling pathway has been shown to mitigate blood–brain barrier (BBB) disruption after cerebral ischemia/reperfusion injury [32] and also following traumatic brain injury (TBI), phosphorylation levels of both Lats1 and YAP1 were upregulated in injured regions, and inhibition of Lats1 not only downregulated the level of p-YAP1, but also attenuated neuronal apoptosis and neurological impairment [33]. Additionally, YAP1 was one of the top 10 hub genes identified for AD [34]. More importantly, YAP1 promotes adult hippocampal neural stem cell activation, while dysregulated YAP1 activity leads to repression of hippocampal neurogenesis [35]. These abundant data clearly demonstrate that YAP1 is crucial for neurodegenerative diseases and therefore it is important to identify the exact molecular mechanisms in the brain-relevant cells.

Here, we generated SH-SY5Y neuroblastoma cells stably expressing YAP1-GFP and subjected them to a multiplex antibody-based protein quantification method to detect significant alterations in proteins. Interestingly, we found upregulated markers of the Ras homologous guanosine triphosphatase (Rho GTPase)-related proteins that control the cytoskeleton and cell shape, as well as the autophagy–lysosome pathway (ALP). On the other hand, we found a significant reduction of senescence marker p16INK4a, which was downregulated by more than 50% in YAP1-GFP-expressing SH-SY5Y cells relative to control cells.

## 2. Materials and Methods

### 2.1. Chemicals and Antibodies

The components of the cell lysis buffer, such as sodium orthovanadate (cat # 450243), dithiothreitol (cat # D9779), and the protease inhibitor cocktail (cat # P8340), were all purchased from Sigma Aldrich (St. Louis, MO, USA). The protease inhibitor microcystin-LR (cat# 475815) was purchased from Calbiochem-Millipore (Temecula, CA, USA). The Nonidet-P40 substitute (cat # M158) to prepare lysis buffer was obtained from Amresco (Solon, OH, USA). The stock NuPAGE™ LDS Sample Buffer (4×) was purchased from Fisher Scientific (cat # NP0007). The prestained protein ladder, PageRuler™, with a range of markers from 10 to 180 kDa (cat # 26617), and the SuperSignal™ West Pico PLUS Chemiluminescent Substrate (cat # 34578) for detection were purchased from Thermo Fisher Scientific, Waltham, MA, USA. The rabbit polyclonal antibodies against YAP1 (cat # 4912) were from Cell Signaling. The rabbit polyclonal antibodies ARHGEF1 (cat # A4274) and NEDD9 (cat # A2521) were purchased from Abclonal (Woburn, MA, USA). Monoclonal beta-actin antibody (C4) (cat # sc-47778) and LC3B antibody (cat # sc-398822) were from Santacruz. The secondary antibodies, such as peroxidase-conjugated AffiniPure goat anti-mouse (Code # 115-035-146) and goat anti-rabbit (Code # 111-035-144) IgG (H+L), were purchased from Jackson ImmunoResearch Laboratories (West Grove, PA, USA). For immunoblot analysis, a 5% Americanbio Inc non-fat dry milk (cat #NC0115668, Fisher Scientific (Waltham, MA, USA) prepared in tris-buffered saline (TBS) with 0.1% Tween-20 (TBS-T) was used to dilute all the primary antibodies, while the secondary antibodies were diluted directly in the 1× TBS-T buffer.

### 2.2. Generation of SH-SY5Y Stable Cells Expressing YAP1-GFP and Cell Lysate Preparation

We cultured the immortalized human neuroblastoma cell line SH-SY5Y (cat # CRL2266, ATCC) in DMEM/F12 medium and infected only once with Lenti ORF particles, human YAP1 (mGFP-tagged) transcript variant 1 (cat # RC225864L4V, Origene, Rockville, MD, USA), and generated stable cells expressing YAP1-GFP with puromycin selection at 5.0 µg per milliliter for two weeks. Control SH-SY5Y cells expressing mGFP alone were generated by infecting control Lenti GFP particles (cat # PS100093V, Origene). Equal cell numbers of both YAP1 and mGFP control stable cells were plated onto 10 cm plates and were maintained. On day three of culture, the cell lysates were prepared in 1%NP40 buffer, and protein concentrations were determined by the BCA method.

### 2.3. Immunocytochemical Staining of Cell Lines and Human Neurons

We purchased astrocytoma (cat# CCF-STTG1), HMC3 (cat # CRL-3304), SH-SY5Y (cat # CRL2266), and Ntera-2 (NT2) cells (cat # CRL-1973) from ATCC (Manassas, VA, USA) as cellular models of astrocytes, microglia, neuroblastoma, and neurons, respectively. Human neurons (HNs) isolated from the human brain were obtained from ScienCell (cat # 1520). The neurons were cultured in neurobasal medium (cat # 21103049, Thermo Fisher) containing 2% B-27 supplement (cat# 1-7504-044, Life Technologies, Grand Island, NY, USA), glutamine (cat# 25030-081, Life Technologies), sodium pyruvate (cat# 11360, Life Technologies), and penicillin/streptomycin (50 units/mL penicillin, 50 μg/mL streptomycin, cat # 30-002-C1, Corning, Glendale, AZ, USA) and plated on to a sterile coverslip in the 12-well plates. Half of the growth medium was changed twice weekly. The neurons were grown until 18 days in vitro (18DIV) and were subjected to immunocytochemical staining as follows. Briefly, cells or neurons were washed three times with 1× phosphate-buffered saline (PBS), fixed with 4% paraformaldehyde (PFA) for about 10 min, followed by three washes with 1× PBS and permeabilization with tris-buffered saline (TBS) with 0.1% Tween 20 detergent (TBST) and then blocked with an in-house-prepared blocking solution (normal donkey serum, 1%; BSA, 3%; gelatin, 1%; Triton X-100, 0.2%; saponin, 0.2%) for 30 min. Immunostainings were performed by incubating cells with YAP antibody (cat # 4912, Cell Signaling) at a 1:100 dilution for overnight. The next day, the cells were incubated with Alexa Fluor 488-conjugated anti-rabbit IgG secondary antibody for 1 h, followed by mounting with 4′,6-diamidino-2-phenylindole (DAPI) containing Fluormount-G (Southern Biotech, Birmingham, AL, USA) to visualize the nuclei, and images were captured in a BZX700 All-in-One microscopy system (Keyence Corp, Itaska, IL, USA).

### 2.4. Antibody-Based Multiplex Proteomic Screening

To identify molecules/pathways responsible for YAP1 signaling, since proteomics is a very powerful tool for discoveries [36,37], we screened 8000 human proteins using multiplex arrays from Ray Biotech (cat # AAH-BLM-8000) that uses biotin-label based antibody arrays, a glass slide array (Human L-507). To do this, lysates from both control and YAP1-overexpressed cells with an equal protein quantity of 20 µg were sent to Ray Biotech for further processing. The antibody array-based proteomic analysis was carried out based on a single control and a single YAP1-overexpressing sample. Therefore, it should be noted that the array serves as an exploratory screening rather than a statistically powered differential proteomic analysis. The samples were biotinylated and incubated with HRP-conjugated streptavidin and detection buffers. The detection was done by the chemiluminescent imaging system. This was followed by densitometric quantification. After normalization was done against both positive controls and the intensity, raw numerical data were generated. The Ward Hierarchical Cluster method was used to set the distance between clusters to the ANOVA sum of squares between the two clusters summed over all the variables. At each generation, two clusters from the previous generation were merged to reduce the within-cluster sum of squares over all partitions. The sums of squares are easier to interpret when they are divided by the total sum of squares to give the proportions of variance (squared semi-partial correlations). This method joins clusters to maximize the likelihood at each level of the hierarchy under the assumptions of multivariate normal mixtures, spherical covariance matrices, and equal sampling probabilities.

### 2.5. Bioinformatics Analysis and Heatmap Generation

We utilized Ray Biotech’s Biostatistics and Bioinformatics services to analyze the proteomic data. All the analyses were conducted in the R programming language V4.2.3 [38]. Briefly, data were filtered by excluding all the biomarkers demonstrating no variation among all the samples, and then the standardized data were plotted in a heatmap in which the different colors represent biomarker expression levels with hierarchical clustering by Euclidean distance. For the heatmap generation, the 8000 biomarkers of targets in two samples were clustered into 100 groups by Euclidean distance after scaling and centering. In this analysis, we calculated the ratio between control and YAP1 overexpressed samples for each target with the data normalized with positive values, and then transformed the ratios into Z-scores. The targets with Z-scores > 1.96 or <−1.96 were considered as differentially expressed. The KEGG pathway enrichment and GO term enrichment were conducted as an over-representation evaluation on the differentially expressed biomarkers involving various KEGG pathways/GO terms, with all the targets measured as background. A Gene Set Enrichment Analysis using all the ratios of biomarkers/targets was conducted, too. The Pathway/GO over-representation and GSEA analysis were implemented with the R package cluster Profiler (v4.2.3; R Core Team 2021) [39].

### 2.6. Quantification of Proteins by Western Blotting

The cell lysates from control and YAP1-GFP-expressing SH-SY5Y cells were prepared in a 1%NP40 buffer that contained a complete protease inhibitor mix supplemented with sodium vanadate and microcystin to inhibit several classes of ATPases, protein tyrosine phosphatases, and other enzymes. Following rigorous vortexing, the cell lysates were centrifuged and mixed with equal amounts of NuPAGE™ LDS sample loading buffer and subjected to SDS-PAGE electrophoresis exactly as described previously [40,41,42]. The proteins were then transferred onto nitrocellulose membranes, blocked with 5% milk prepared in 1% TBS-T buffer, and incubated overnight with primary antibodies at 1000–2000 dilution, followed by 1–2h incubation with HRP-conjugated anti-rabbit or anti-mouse secondary antibodies in 1× TBS-T buffer. The protein signals were detected at different exposure times following incubation with the SuperSignal West Pico Chemiluminescent Substrate. The freely available ImageJ software (https://imagej.net/ij/index.html, access date 13 May 2026) from NIH was used for the quantification of immunoblot signals. All blots were re-probed with an actin antibody as a loading control, and the actin protein levels were used for normalization of proteins. The protein levels are shown as percentage change or fold change from control cells.

### 2.7. Statistical Analysis

Statistical analyses for changes in immunoblot protein levels were performed using the GraphPad Prism Software version 9.5.1 (GraphPad, San Diego, CA, USA). For comparisons in the levels of ARHGEF1, NEDD9, p16INK4a, and LC3 between two groups, such as control and YAP1-overexpressing cells, Student’s *t*-test with two-tailed parameters was used. Data presented are the mean ± standard error of the mean (SEM) and were considered significant only if *p* < 0.05. * indicates *p* < 0.05, ** indicates *p* < 0.01 and *** indicates *p* < 0.001.

## 3. Results

### 3.1. Neurons and Other Brain Cell Types Express YAP1 Protein

**Figure 1:**
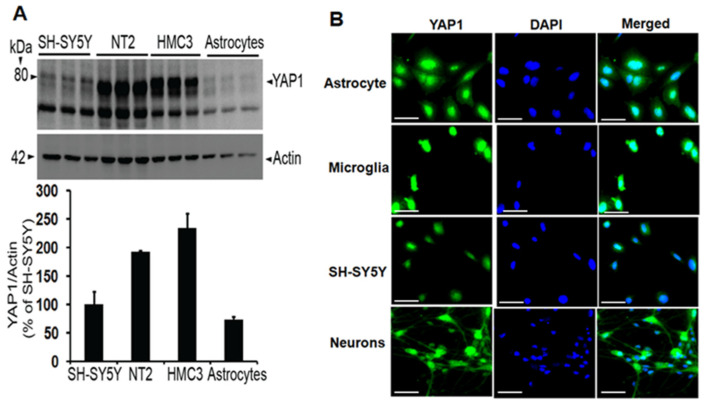
(**A**)YAP1 protein is expressed in different cell types relevant to the brain, such as astrocytes (CCF-STTG1), microglia (HMC3), neuroblastoma (SH-SY5Y), and NT2 cells, as demonstrated by immunoblotting. Data are mean ± SEM (*n* = 3). (**B**) Images show immunocytochemical evidence of YAP1 expression (in green) and DAPI-stained nuclei (in blue) in different cell types and human neurons. Scale: 20 µm.

YAP1 expression has been widely studied in non-neuronal cells, but whether YAP1 is also expressed in different cell types of the brain has not been thoroughly investigated. Therefore, we quantified actin-normalized YAP1 protein levels and analyzed relative expression among the cell line models of astrocytes (CCF-STTG1), microglia (HMC3), neuroblastoma (SH-SY5Y), and neuronal cell line (NT2). Relative comparison among the cell types showed that HMC3 microglia expressed the highest level of YAP1 followed by neuronal NT2 cells, SH-SY5Y neuroblastoma cells and astrocytes (Figure 1A). To increase rigor, we also confirmed YAP1 expression with an immunocytochemical method using the same cell lines by staining with anti-YAP1 antibody (Figure 1B). Thus, we have shown that the YAP1 protein is expressed in multiple types of brain cells by both immunoblots and immunocytochemistry. Since YAP1 expression in neurons is crucial, we also cultured human neurons until 22DIV and confirmed YAP1 protein expression in the human neurons, as shown in Figure 1B. This data suggests that the YAP1 protein is expressed in human neurons.

### 3.2. YAP1 Protein Expression Is Downregulated in the Nuclear Fractions of Alzheimer’s Brains

**Figure 2:**
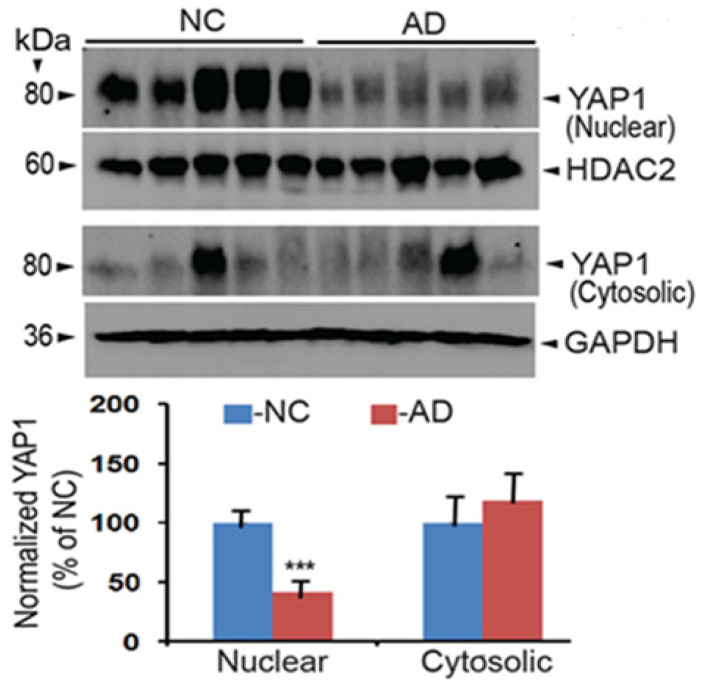
YAP1 protein levels are significantly downregulated in the nuclear fractions but not the cytosolic fractions of the AD brains relative to NC brains. Brain lysates were immunoblotted, and protein levels were quantified by normalizing to HDAC2 levels for nuclear fractions and to GAPDH levels for cytosolic fractions. Data are mean ± SEM (*n* = 5 per group). *** *p* < 0.001 by *t*-test.

A previous study showed that intracellular Aβ sequesters YAP1 to the cytoplasm, thereby depriving YAP1 of the nucleus as demonstrated in mild cognitive impairment (MCI) and AD patients, which can lead to neuronal necrosis due to reduced YAP1 function [43]. This implies that the YAP1 protein may be reduced in AD brains. To test this possibility, the AD and normal control (NC) brain tissues (hippocampus) were obtained (Table 1) from the “Harvard Brain Tissue Resource Center,” which is supported in part by PHS grant number R24MH068855. As YAP1 is known to be present in both the nucleus and the cytosol, we separated cytosolic and nuclear fractions using NEPER ™ Nuclear and Cytoplasmic Extraction Reagents from Thermo Fisher (cat # 78835). The nuclear and cytoplasmic extracts were immunoblotted, and YAP1 protein levels were quantified using the YAP1 antibody. To increase rigor, the YAP1 protein levels in the nuclear extracts were normalized to HDAC2 levels used as a nuclear marker, while cytoplasmic levels were normalized to GAPDH protein levels used as a marker of cytoplasmic proteins. Thus, any minor errors in loading the protein samples were taken care of with normalization of marker proteins quantified by reprobing the same blots used for YAP1 protein quantification. As shown in Figure 2, YAP1 protein levels were downregulated by 41% in AD brains relative to NC brains, which was highly significant (***, *p* < 0.001, *n* = 5/group by *t*-test). On the other hand, although a 19% upregulation was seen in the cytoplasmic extracts of AD brains, it was found to be statistically insignificant. This clearly suggests that the YAP1 protein is selectively reduced in the nuclear fractions of AD brains, and this reduction is expected to reduce co-transcriptional activity of YAP1 in the AD brains.

### 3.3. Multiplex Proteomic Screening Revealed Alterations in Specific Proteins and Pathways

**Figure 3:**
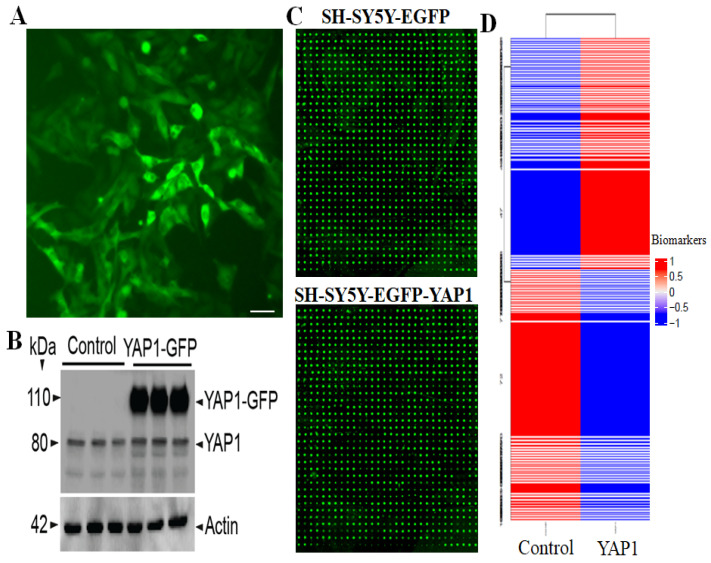
Antibody-based multiplex microarray screening of 8000 proteins revealed alterations of specific proteins. (**A**) SH-SY5Y cells expressing YAP1-GFP. Scale, 50 µm. (**B**) YAP1-GFP expression supported by immunoblot. (**C**) Examples of glass slide luminescence. (**D**) Heatmap of control and YAP1 biomarkers in 8000 samples. The data were imported to JMP Genomics 10.2 for Hierarchical Cluster analysis.

To identify differentially altered proteins due to YAP1 expression, the ratio between biomarker values from the two samples was calculated as YAP1/Control. A small number (0.01) was added to the biomarker values of YAP1/Control when either of these two values was zero. The Z-score of the ratios is calculated as (ratio-mean)/(standard deviation of the ratios). The targets with a higher absolute Z-score (>1.96) were considered as differentially expressed. To generate a heatmap, the 8000 targets were clustered into 100 groups by Euclidean distance of biomarker values. A typical example of YAP1-GFP-expressing SH-SY5Y cells is shown in Figure 3A. The confirmation of YAP1 overexpression is shown in Figure 3B, which shows YAP1-GFP fusion protein and endogenous YAP1, where YAP1-GFP is expressed only in stable cells and not in controls. Figure 3C shows array scan images for the control and YAP1-expressing cells. Figure 3D also shows the significant clusters in the heatmap obtained after importing data into JMP Genomics 10.2 hierarchical cluster.

### 3.4. Gene Ontology (GO) Over-Representation Analysis

**Figure 4:**
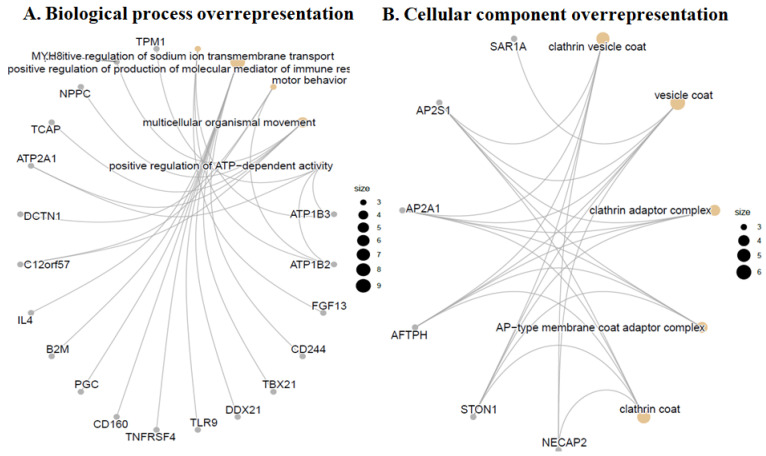
YAP1 expression increased markers of synaptic endocytosis and ATP-dependent activity. (**A**) Gene ontology (GO) biological process over-representation analysis with 283 differentially expressed biomarkers between YAP1 and control samples. (**B**) GO cellular component over-representation analysis with 283 differentially expressed biomarkers between control and YAP1-expressing SH-SY5Y cells.

**Figure 5:**
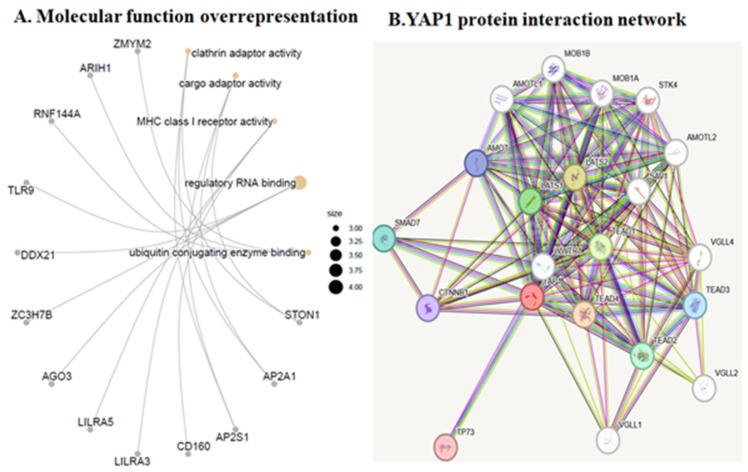
(**A**) GO molecular function over-representation analysis with 283 differentially expressed biomarkers between YAP1 and control samples. (**B**) Interaction network of human YAP1 protein as analyzed by STRING.

Functional enrichment analysis of 283 differentiallyexpressed biomarkers between control and YAP1-expressing samples on biological process and GO terms revealed upregulated markers of synaptic endocytosis and ATP-dependent activity. As shown in Figure 4A, markers of positive regulation of sodium ion transmembrane transport and production of molecular mediators of immune response, motor behavior, synaptic vesicle endocytosis, ATP-dependent activity, and presynaptic endocytosis markers were overrepresented in the YAP1-expressing cells relative to control cells. GO cellular component over-representation analysis revealed upregulated markers of clathrin vesicle coat and adapter complex, AP-type membrane coat adaptor complex, cation-transporting ATPase complex, ATPase-dependent transmembrane transport complex and proteasome regulatory particle in YAP1-expressing neuroblastoma cells relative to control cells as shown in Figure 4B. The GO molecular function over-representation analysis also revealed upregulated markers of clathrin adaptor activity, ubiquitin-conjugating enzyme binding, GTPase activator activity, exonuclease activity, active with either ribo- or deoxyribonucleic acids and producing 5’-phosphomonoesters and ubiquitin-like protein conjugating enzyme binding in the YAP1-expressing neuroblastoma cells relative to control cells as shown in Figure 5. Further, KEGG pathway analysis revealed anover-representation of markers of insulin secretion, bile secretion, thyroid hormone secretion pathway, efferocytosis and autoimmune thyroid disease in the YAP1 cells relative to control cells. Thus, cellular and molecular pathway analysis revealed upregulated biomarkers of synaptic vesicle and clathrin adaptor proteins following YAP1 expression in cells.

### 3.5. Ward Hierarchical Cluster Analysis Revealed an Increase in Specific Markers of Endocytosis and Actin Cytoskeleton

Table 2 provides a list of proteins upregulated by more than 1-fold by YAP1 expression, with the highest Z-scores. The top ten upregulated proteins and their extent of increase include NECAP2 by 6.19-fold, HRASLS2 by 3.51-fold, C1QTNF6 by 3.29-fold, MLANA by 2.96-fold, NENF by 2.07-fold, AAMDC by 2.02-fold, PSMG3 by 1.91-fold, CCDC124 by 1.85-fold, CCDC40 by 1.83-fold, and ATP1B4 by 1.81-fold. Additionally, fwe utilized STRING, a core data resource as designated by the Global Biodata Coalition and ELIXIR [44], to study the YAP1 protein interaction network as shown in Figure 5B. The major interacting proteins are LATS2 (Identifier: ENSP00000372035), TEAD2 (Identifier: ENSP00000472109), TP73 (Identifier: ENSP00000367545), AMOT (Identifier: ENSP00000361027) and SMAD7 (Identifier: ENSP00000262158) (Figure 5B).

### 3.6. Pathway Enrichment Analysis by ShinyGo

**Figure 6:**
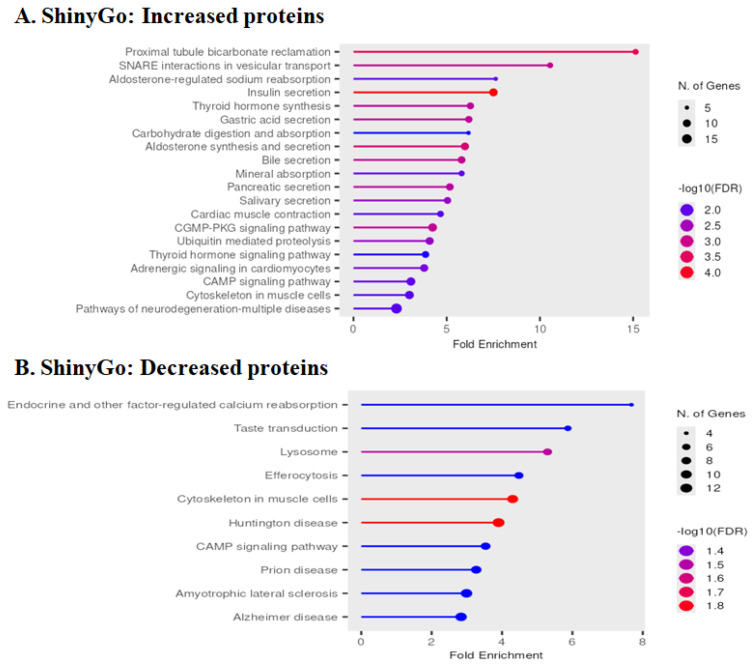
The bar graph shows GO analysis results of the increased (**A**) and decreased proteins (**B**) due to YAP1 expression using ShinyGO 0.85.1. Sets of nonredundant, significant GO enrichment terms are displayed to show the foldenrichment and the number of proteins matched to each term. The fold-enrichment values are displayed with the highest on top, and the shade of each bar reflects the number of proteins found in a given GO category.

GO enrichment analysis of YAP1-altered proteins using ShinyGO 0.85.1 [45] additionally revealed GO term enrichment for SNARE interactions in vesicular transport, aldosterone, and insulin secretion regulation (Figure 6A). Also as predicted, proteins involved in the cytoskeleton, cardiac signaling, and pathways of neurodegeneration were also found to be enriched in the upregulated proteome set. Among the downregulated proteome, calcium reabsorption regulation, cytoskeleton, lysosome, efferocytosis, and multiple neurological diseases were significantly represented (Figure 6B). Detailed differentially altered proteome results are summarized in Table 2 and Table 3.

### 3.7. Validation of Multiplex Array Results by Immunoblots

**Figure 7:**
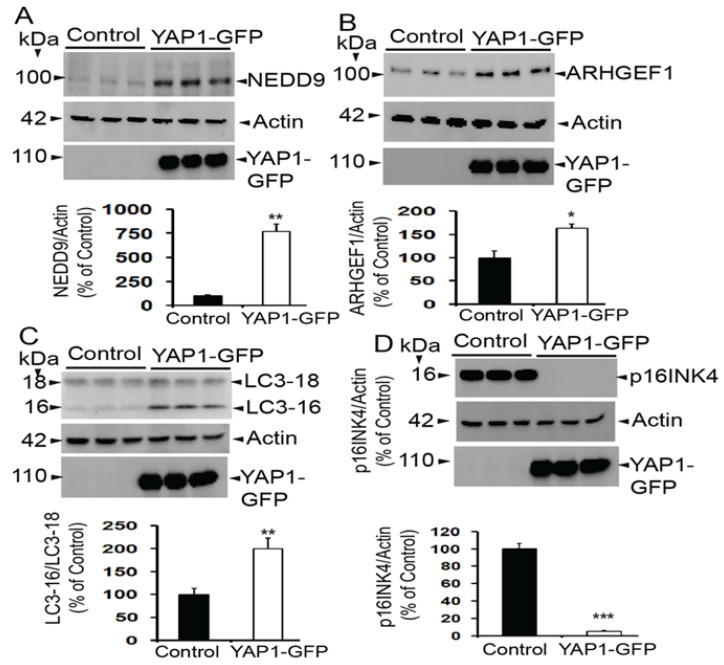
Evidence of increased levels of (**A**) NEDD9 and (**B**) ARHGEF1 inYAP1-GFP-expressing SH-SY5Y cells when compared to control cells by immunoblotting. (**C**) The LC3-16/LC3-18 ratio was also increased, indicating YAP1 activates autophagy. However, levels of (**D**) p16INK4a, a marker of senescence, were almost completely abolished. Data are mean + SEM, *n* = 3/group. *, *p* < 0.05, **, *p* < 0.01, ***, *p* < 0.001 by *t*-test.

Although multiplex antibody array-based proteomics is a very powerful tool for discoveries [36,37], the cross-reactivity of antibodies may limit assay performance and thus may result in inaccurate and even false results, and wrong conclusions. Thus, to ensure the accuracy of the results, at least some crucial analytes should be validated by standard methods. Therefore, here we used the standard immunoblot method to confirm the upregulated protein levels of some important proteins. Similar to the array results, we found significantly upregulated levels of NEDD9 (Figure 7A) (by 7.64-fold) compared to controls (**, *p* < 0.01 by *t*-test) and ARHGEF1 (Figure 7B) by 0.64-fold (*, *p* < 0.05 by *t*-test) relative to controls, thereby validating the proteomic array results.

### 3.8. YAP1 Also Changes Expression of LC3 and p16INK4a

Additionally, we found upregulated levels of LC3, a marker of autophagosomes; both the 16 Kda and the 18 kDa forms were upregulated. We found the ratio of LC3-16/LC3-18 was upregulated by 1-fold (**, *p* < 0.01 by *t*-test) when compared to the controls (Figure 7C). We selected LC3 because its ratio is a very reliable marker of autophagosome formation and autophagy [46]. This increased ratio of LC3 suggests that YAP1 expression increases autophagy flux, and therefore, we may interpret that YAP1 enhances autophagy. Interestingly, as shown in Figure 7D, we also found a drastic 95% reduction in the expression levels of p16INK4a (***, *p* < 0.001 by *t*-test), a widely used and validated marker of cellular senescence [47,48,49]. This finding is consistent with other investigators who have proposed an anti-senescence role for YAP1 in multiple tissues, including astrocytes [18,19,20,21,23,24].

## 4. Discussion

YAP1 is a key member of the Hippo signaling pathway, which can regulate organ size as it can sense various types of mechanical cues, including membrane tension, cell stretching, and cell stiffness via actin stress fibers. Although YAP1 is known to exert positive effects on diverse brain-related functions, its potential mechanism remains incompletely understood. Therefore, in the present study, we expressed YAP1 in the SH-SY5Y neuroblastoma cells, and the lysates were subjected to antibody-based multiplex array scan, and detected 283 differentially expressed proteins. While the results supported the previously known function of YAP1 on the actin cytoskeleton through RhoGTPase-related proteins, the screening also identified novel YAP1 mediators for tissue regeneration, such as NENF and NEDD9, and for anti-senescence effects through p16INK4a.

As the function of a given protein depends on its expression pattern, we first addressed whether the YAP1 protein is detectable in different cell types of the brain. We found its expression in all cell types examined, i.e., microglia, astrocytes, neuroblastoma, and neuronal cell lines. Multiple previous studies have also shown YAP1 expression in the mouse brain and cultured cells. Thus, Xu et al., 2021 [22] showed YAP1 expression mainly in the astrocytes by immunostainings of the hippocampus in young and old mice. Huang et al., 2020 [50] also did not detect YAP1 expression in microglia and neurons but showed high expression in the astrocytes within the mouse brain by immunohistochemistry. Yu et al., 2020 [51] also detected YAP1 by immunofluorescence staining mainly in the astrocytes, but there was almost no YAP1 expression in neurons and microglia of spinal cord-derived cell cultures, which was consistent with another study [52]. However, in another study, YAP1 was shown to be expressed in NeuN-positive neurons, Iba1-positive microglia, and GFAP-positive astrocytes in the spinal cord as well as MAP2-positive-dendrites in mixed spinal cord-derived neuronal cultures [14]. Our results are consistent with this study, with the positive demonstration of YAP1 in these multiple cell types. Importantly, since YAP1 expression in human cells is crucial for any translational applications, we also found YAP1 expression in cultured human neurons. Our result is also consistent with multiple previous studies, which demonstrated that YAP1 is predominantly expressed in neurons [29,53,54]. Thus, there are differences in the reporting of YAP1 expression in neurons.

Because YAP1 was one among the top 10 hub genes identified for AD [34], we were also interested in verifying whether YAP1 protein levels are altered in AD brains and found selective loss of YAP1 protein in the nuclear but not cytoplasmic fractions of AD brains relative to normal controls. In another study, quantification of signal intensities of nuclear YAP staining in neurons confirmed reduced YAP1 levels in three mild cognitive impairment (MCI) and three symptomatic AD patients [43]. Interestingly, the study found that reduced nuclear YAP1 is due to sequestration of YAP1 into the cytoplasm by Aβ aggregates in cortical neurons of AD and MCI patients, which was also confirmed in cortical neurons of 5xFAD mice and human mutant APP-KI mice [43]. YAP1 has also been shown to be downregulated and inactivated in senescent astrocytes, both in the cultured senescent astrocytes and hippocampal astrocytes of the aging as well as AD model mice, suggesting that YAP1 may play an important role in astrocyte senescence [22]. There is also evidence that YAP1 is reduced both in vivo and in vitro in the hippocampus of both aged C57BL/6J mice and the SAMP8 mouse model of AD through Hippo pathway activation [29]. Thus, our demonstration of reduced nuclear YAP1 in AD brains by immunoblots supports multiple other studies.

Among the upregulated proteins, the important ones are NECAP2, which is the highest upregulated protein and plays an essential role in clathrin-mediated endocytosis [55], and a specific genetic variation (rs6859) in the NECTIN2 gene, which encodes NECAP2, which has been linked to increased levels of phosphorylated tau (pTau), a key biomarker for AD, and is associated with AD risk [56]. Another upregulated protein is NENF, also known as Neudesin, which is a protein with multiple functions, primarily acting as a neurotrophic factor, and it promotes neuronal survival, enhances cell proliferation, and neurogenesis in neural progenitor cells [57,58], exactly mimicking YAP1 function. ARHGEF1, also known as p115 RhoGEF, is a protein that functions as a guanine nucleotide exchange factor (GEF) for the RhoA GTPase and is crucial for dendritic spine regulation [59]. Another upregulated protein, NEDD9, also known as HEF1 or CASL, is a scaffolding protein that affects neuronal cell number and synaptic connections through its role in neurite formation [60], and is genetically associated with AD [61,62,63]. Importantly, reduced NEDD9 expression or function may contribute to the neuropathological changes observed in AD [64], such as the loss of neurons and synapses. Another important protein upregulated is DCTN6 (dynactin subunit 6), crucial for the function of the molecular motor dynein in microtubule-based intracellular transport, and its deficiency enhances aging-associated dystrophic neurite formation in mouse brains [65]. It is interesting to note that upregulated proteins such as NEDD9 and NENF by YAP1 recapitulate essential function of YAP1 in terms of cell proliferation and neurogenesis in neural progenitor cells [35]. Thus, these proteins may also be responsible for YAP1-mediated healthy aging and tissue regeneration.

The pathway enrichment analysis by both KEGG and ShinyGo mainly identified clathrin-and cargo adaptor activity, SNARE interactions, cytoskeleton, and neurodegeneration pathway molecules. Also, STRING analysis identified validated YAP1-interacting proteins, including LATS proteins, which play a pivotal role in organ size control, TEADs, which also play a crucial role in organ size regulation, TP73, involved in apoptotic response to DNA damage, AMOT, involved in tight junction regulation, and SMAD7, which antagonizes TGF-β signaling. Overall, these results indicate that YAP1 regulates molecules crucial for the cytoskeleton, organ size, DNA damage response, and cell growth.

Another important finding from our study is a drastic reduction in the levels of senescence marker p16INK4a, a primary marker of cellular senescence [66], after YAP1 overexpression in the SH-SY5Y neuroblastoma cells. While we are the first to demonstrate reduced p16INK4a after YAP1 overexpression, upregulated p16INK4a has been shown after conditional knockout of YAP1 in astrocytes, which significantly promoted premature senescence of astrocytes [22]. Another study also showed increased p16INK4a mRNA expression following inactivation of YAP1 [67]. These studies, taken together with our results, suggest that YAP1 overexpression reduces while YAP1 inactivation increases the senescence marker p16INK4a. In general, two canonical pathways, i.e., the p53/p21 and the pRb/p16 axes, have been implicated in cellular senescence induced by various insults. While our study shows involvement of YAP1 in the pRb/p16 pathway, several previous studies have also implicated YAP1 in the p53/p21 pathway. For instance, YAP can directly bind to the p53 gene promoter and upregulate p53 expression, and YAP/Hippo and p53 pathways functionally and physically interact to govern cell-fate decisions [68]. Also, YAP–TEAD counteracts the pro-apoptotic side of p53 in senescent cells by transcriptionally repressing DDIT4 production [69]. Indeed, in another study, a conditional knockout of YAP1 has been shown to increase senescence-associated β-galactosidase activity, as well as several senescence-associated genes, including p16, p53, and NF-κB [22]. This evidence implies that YAP1 is involved in both p53/p21 and the pRb/p16 pathways in regulating senescence. Thus, the anti-senescence effect of YAP1 may be crucial for its regenerative functions, given that YAP1 activity declines during physiological aging, and sustaining YAP1 function rejuvenates old cells [25].

### Limitations and Future Directions

The antibody array-based proteomic analysis was carried out based on a single control and a single YAP1-overexpressing sample. Therefore, it should be noted that the array serves as an exploratory screening rather than a statistically powered differential proteomic analysis. Also, for validation studies, we picked proteins based on their significance to neuronal regeneration and neuroprotective properties rather than their extent of increase. Future studies will focus on the validation and functional significance of other essential proteins altered by YAP1 expression. Also, although an increased ratio of LC3 suggests that YAP1 expression may increase autophagy flux, multiple other supportive data are needed for this interpretation. Also, the role of YAP1 in neuronal survival, regeneration, synaptic function, or stress resistance and its potential mechanism through TEAD and Hippo signaling pathway will be addressed in future studies. Overall, since we used array data in this study, it is mostly hypothesis-generating in nature and therefore is mainly descriptive rather than having any mechanistic conclusions.

## 5. Conclusions

In conclusion, we have shown that YAP1 protein is expressed in multiple cell types of the brain, and that YAP1 protein is selectively reduced in the nuclear fractions of AD brains, which may be responsible for neurodegeneration due to loss of protection against neuronal death. Additionally, our multiplex antibody-based proteomic study has identified novel proteins such as NENF and NEDD9, which may account for the tissue-regenerative effects of YAP1.

## Figures and Tables

**Table 1:** Demographics of the non-diseased controls (NC) and AD brain tissue donors.

Numbers	Gender (M/F)	Age (Y)	Avg. Age (Y)	PMI (h)	Avg. PMI (h)
NC-1	F	58	78.2 ± 11.88	26.6	25.48 ± 4.23
NC-2	F	77		28	
NC-3	F	84		18.37	
NC-4	F	86		29.18	
NC-5	M	86		25.28	
AD-1: Braak-3	F	85	89.6 ± 5.98	17.67	16.46 ± 6.15
AD-2: Braak-3	F	97		20.66	
AD-3: Braak-3	F	87		22.32	
AD-4: Braak-6	M	95		15	
AD-5: Braak-6	M	84		6.66	

The gender distribution of male/female (M/F), age in years (Y), and the postmortem interval (PMI) for tissue collection in hours (h); NA-not available.

**Table 2:** Top 25 increased proteins by YAP1 overexpression with Z-scores. The ratio between biomarker values from two samples was calculated as YAP1/CONT. A small number (0.01) was added to the biomarker values of YAP1 and CONT when either of these two values was zero. The Z-score of the ratios is calculated as (ratio-mean)/(standard deviation of the ratios).

Biomarker	Control	YAP1	Ratio	Z-Score
NECAP2	2842.2	17,595.85625	6.1909282	24.24106
HRASLS2	3963.93	17,883.00124	4.5114321	16.40454
C1QTNF6	7172.005	30,784.1465	4.2922651	15.3819
MLANA	7971.755	31,577.68934	3.9611967	13.83714
NENF	8540.74	26,220.81979	3.0700876	9.679228
AAMDC	11,441.935	34,569.90192	3.0213335	9.451742
PSMG3	3612.53	10,530.97212	2.9151238	8.956168
CCDC124	11,260.29	32,188.0279	2.8585434	8.692164
CCDC40	11,606.205	32,901.31207	2.8348036	8.581395
ATP1B4	8582.515	24,197.27492	2.8193688	8.509376
SGCB	3575.35	9637.037696	2.695411	7.930989
AFAP1L2	11,417.26	29,729.79165	2.603934	7.504158
TBC1D10A	4897.34	12,600.11439	2.5728486	7.359114
ARHGEF1	11,990.095	30,405.01054	2.535844	7.18645
CEACAM21	12,934.665	32,600.91571	2.5204298	7.114528
NEDD9	7149.23	17,649.76542	2.4687645	6.873458
CCNA1	9850.31	23,938.24687	2.4302024	6.693527
C5orf22	12,640.265	30,524.52924	2.4148647	6.621961
ARHGEF6	9403.465	22,519.34664	2.3947924	6.528304
ATP1B3	13,777.05	32,787.23206	2.3798442	6.458556
ADRM1	12,350.02	28,773.95093	2.3298708	6.22538
OVGP1	6283.445	14,499.13167	2.3075131	6.121059
ARHGAP12	13,198.675	30,350.94912	2.2995452	6.083881
AFF4	11,142.845	25,560.02972	2.2938513	6.057314
INHA	2289	5226.75186	2.2834215	6.008648

**Table 3:** Top 25 decreased proteins by YAP1 overexpression with Z-scores. The ratio between biomarker values from two samples were calculated as YAP1/CONT. A small number (0.01) was added to biomarker values of YAP1 and CONT when either of these two values was zero. The Z-score of the ratios is calculated as (ratio-mean)/(standard deviation of the ratios).

Biomarker	Control	YAP1	Ratio	Z Score
**BMP8A**	3519.205	1530.569321	0.434919057	−2.616455578
**SOX17**	256,997.68	111,422.7754	0.433555569	−2.622817607
**DNMT3A**	5654.15	2402.735552	0.424950798	−2.662967431
**AP2S1**	27,067.66	11,168.97539	0.412631731	−2.720448138
**CANT1**	6108.575	2507.032767	0.410412046	−2.730805174
**GRM3**	13,185	5372.991016	0.407507851	−2.744356136
**BTF3L4**	111,380.13	44,613.8194	0.400554564	−2.776800141
**CD99**	5207.55	2081.721646	0.399750679	−2.780551066
**Cytochrome C (d)**	83,073.77	32,204.89888	0.387666274	−2.836936841
**Nestin**	127,685.16	49,437.34844	0.38718163	−2.839198185
**BTN3A1**	5298.35	1982.393163	0.374152927	−2.899990052
**ANTXR2**	7864.435	2864.704529	0.364260691	−2.94614718
**ACADS**	28,985.865	10,499.07826	0.362213729	−2.955698293
**TPM1**	104,427.55	36,056.11547	0.345273977	−3.034739099
**CADM4**	6209.25	2137.062241	0.344173973	−3.039871712
**ZFYVE19**	10,713.51	3505.75766	0.32722774	−3.118942756
**BBS2**	5735.075	1631.819504	0.284533246	−3.318155063
**CASP1**	10,737.58	3001.144668	0.279499167	−3.341644049
**CEP152**	27,038.09	7491.05731	0.277055713	−3.353045193
**ADAMTS13**	111,005.525	30,612.66509	0.275776049	−3.359016101
**A2M**	138,485.355	36,955.85697	0.26685751	−3.400629963
**MYH11**	27,475.04	7280.489189	0.264985572	−3.409364416
**MRPS26**	15,175.005	3970.236258	0.26162998	−3.425021591
**RABGAP1**	14,088.765	3513.474486	0.249381297	−3.48217389
**ADAM-9**	239,757.37	35,329.43299	0.14735494	−3.958228385

## Data Availability

All the original data contributions presented in the study are included in the article, and any further inquiries can be directed to the corresponding author.
